# Inhibition of Neuroinflammation in LPS-Activated Microglia by Cryptolepine

**DOI:** 10.1155/2013/459723

**Published:** 2013-05-08

**Authors:** Olumayokun A. Olajide, Harsharan S. Bhatia, Antonio C. P. de Oliveira, Colin W. Wright, Bernd L. Fiebich

**Affiliations:** ^1^School of Applied Sciences, University of Huddersfield, Huddersfield, West Yorkshire HD1 3DH, UK; ^2^Neurochemistry Research Group, Department of Psychiatry, University of Freiburg Medical School, Hauptstrasse 5, 79104 Freiburg, Germany; ^3^Department of Pharmacology, Federal University of Minas Gerais, Avenida Antonio Carlos 6627, 31270-901 Belo Horizonte, MG, Brazil; ^4^School of Pharmacy, University of Bradford, Bradford, West Yorkshire BD7 IDP, UK; ^5^VivaCell Biotechnology GmbH, Ferdinan-Porsche-Strasse 5, 79211 Denzlingen, Germany

## Abstract

Cryptolepine, an indoloquinoline alkaloid in *Cryptolepis sanguinolenta*, has anti-inflammatory property. In this study, we aimed to evaluate the effects of cryptolepine on lipopolysaccharide (LPS)- induced neuroinflammation in rat microglia and its potential mechanisms. Microglial activation was induced by stimulation with LPS, and the effects of cryptolepine pretreatment on microglial activation and production of proinflammatory mediators, PGE_2_/COX-2, microsomal prostaglandin E_2_ synthase and nitric oxide/iNOS were investigated. We further elucidated the role of Nuclear Factor-kappa B (NF-**κ**B) and the mitogen-activated protein kinases in the antiinflammatory actions of cryptolepine in LPS-stimulated microglia. Our results showed that cryptolepine significantly inhibited LPS-induced production of tumour necrosis factor-alpha (TNF**α**), interleukin-6 (IL-6), interleukin-1beta (IL-1**β**), nitric oxide, and PGE_2_. Protein and mRNA levels of COX-2 and iNOS were also attenuated by cryptolepine. Further experiments on intracellular signalling mechanisms show that I**κ**B-independent inhibition of NF-**κ**B nuclear translocation contributes to the anti-neuroinflammatory actions of cryptolepine. Results also show that cryptolepine inhibited LPS-induced p38 and MAPKAPK2 phosphorylation in the microglia. Cell viability experiments revealed that cryptolepine (2.5 and 5 **μ**M) did not produce cytotoxicity in microglia. Taken together, our results suggest that cryptolepine inhibits LPS-induced microglial inflammation by partial targeting of NF-**κ**B signalling and attenuation of p38/MAPKAPK2.

## 1. Introduction

Neuroinflammation is a process which principally involves activation of astrocytes and microglia by inflammatory mediators in various CNS pathologies, including brain inflammation, trauma, ischemia, stroke, brain infections, and neurodegenerative CNS disorders [1, 2], such as multiple sclerosis (MS), Alzheimer's disease (AD), Parkinson's disease (PD), Huntington's disease (HD), and amyotrophic lateral sclerosis (ALS) [3, 4].

Microglia are the resident immune cells in the central nervous system [5] and are now considered to be the primary component of the brain immune system [6]. In neuroinflammation, microglia become activated, undergo a change in morphology, and release various cytotoxic mediators, such as nitric oxide (NO), tumour necrosis factor-alpha (TNF*α*), interleukin-1*β* (IL-1*β*), prostaglandin E_2_, (PGE_2_), and reactive oxygen species (ROS). Overproduction of these mediators has been shown to be toxic to neurons and results in a vicious and self-propagating cycle of neuronal death [7]. Microglial inflammation therefore serves as an important model for investigating potential therapeutic entities for slowing the progression of neuronal cell death in neurodegenerative disorders.

The transcription factor, nuclear factor kappa B (NF-*κ*B), has been shown to control inflammatory responses in microglial cells. Activation of NF-*κ*B is triggered by phosphorylation and subsequent degradation of inhibitor of *κ*B (I*κ*B). This process subsequently leads to translocation of the free NF-*κ*B to the nucleus where it promotes the expression of proinflammatory genes such as the proinflammatory cytokines (TNF*α*, IL-6, IL-1*β*, etc.), cyclooxygenase-2 (COX-2), and inducible nitric oxide synthase (iNOS).

Mitogen-activated protein kinases (MAPK) are critical regulators of pro-inflammatory cytokines (TNF*α*, IL-6 and IL-1*β*) during inflammation [8]. Of the MAPKs, the p38 has been central to anti-inflammatory drug discovery for years due to its importance in the production of the pro-inflammatory cytokines and other mediators [9]. p38 produces inflammation by acting on MAPK-activated protein kinase-2 (MAPKAPK2 or MK2). MAPKAPK2 is stimulated in a wide range of inflammatory conditions and is a potential target for anti-inflammatory drug development [10]. MK2 activation and expression have been shown to be increased in microglial cells stimulated with LPS and gamma interferon [11].

**Figure 1:**
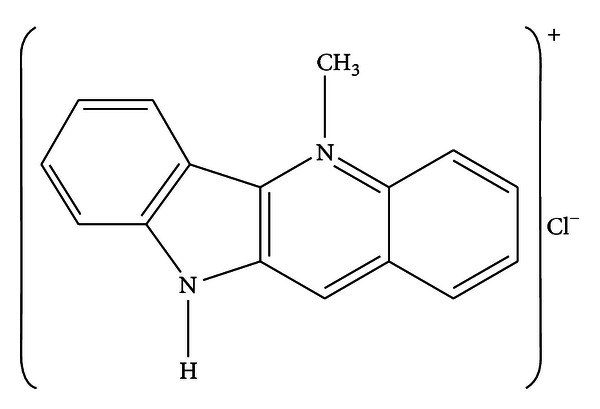


Cryptolepine (5-methyl, 10H-indolo [3, 2-b] quinoline) (Figure 1) is an indoloquinoline alkaloid that is found in abundance in the roots of *Cryptolepis sanguinolenta*, a West African shrub used in the treatment of malaria and other infectious diseases, fevers, pain, and inflammation. Earlier, we showed that this compound produces *in vitro* and *in vivo* anti-inflammatory effects [12, 13]. Although the anti-inflammatory effects of cryptolepine in the periphery have been documented, its effects on cells in the CNS, specifically the microglia, are not known. 

In this study, we sought to investigate the antineuroinflammatory effects of cryptolepine on LPS-activated microglia. We further investigated its molecular mechanisms of action by studying effects of the compound on nuclear translocation of NF-*κ*B, as well as phosphorylation of MAPKs in LPS-activated microglia. As MAPKAPK2 is an immediate downstream substrate of p38 MAPK, we also investigated whether this kinase is also targeted by cryptolepine.

## 2. Methods

### 2.1. Synthesis of Cryptolepine

Cryptolepine (in the hydrochloride salt form) was synthesised by methylation of quindoline as described earlier [12] and based on the methodology of Holt and Petrow [14]. The alkaloid was prepared in sterile water for biological studies and kept at −20°C.

### 2.2. Cell Culture

Primary mixed glial cell cultures were established from cerebral cortices of one-day neonatal Sprague–Dawley rats as described earlier [15]. Forebrains were minced and gently dissociated by repeated pipetting in PBS and filtered through a 70 *μ*m cell strainer (Falcon). Cells were collected by centrifugation (1000 g, 10 min), re-suspended in Dulbecco's modified Eagle's medium (DMEM) containing 10% foetal calf serum (Biochrom AG, Berlin, Germany) and antibiotics (40 U/mL penicillin and 40 *μ*g/mL streptomycin PAA Laboratories, Coelbe, Germany), and cultured on 10 cm cell culture dishes (5 × 10^5^ cells/plate) in 5% CO_2_ at 37°C. Floating microglia were harvested every week (between 2 and 7 weeks) and reseeded into 75 cm^2^ culture flask to give pure microglial cultures. The following day, cultures were washed to remove nonadherent cells, and fresh medium was added. The purity of the microglial culture was >98% as previously determined by immunofluorescence and cytochemical analysis [16].

BV-2 mouse microglia cell line ICLC ATL03001 (Interlab Cell Line Collection, Banca Biologica e Cell Factory, Italy) was cultured in RPMI 1640 (Gibco) supplemented with 10% FBS (Sigma), 2 mM glutamine (Sigma). Cells were split 1 : 5 when they reached confluence using trypsin/EDTA solution in PBS.

### 2.3. Determination of PGE_2_, NO, and the ProInflammatory Cytokines

Rat primary microglial cells were pretreated for 30 min with cryptolepine (2.5 and 5 *μ*M). After prestimulation, cells were incubated with 100 ng/mL LPS for 24 h. Supernatants were then centrifuged at 10,000 g for 10 min. Levels of PGE_2_ in the media were measured by enzyme immunoassay (EIA) (Assay Designs Inc., Ann Arbor, MI, USA) according to the manufacturer's instructions. Nitrite (measure of NO) was determined in supernatants using the Griess assay (Promega) and the proinflammatory cytokines (TNF*α*, IL-1*β*, and IL-6) determined using ELISA kits (Biolegend) according to the manufacturer's instructions.

### 2.4. Western Blot

In order to further establish the mechanisms involved in the action of cryptolepine, experiments were conducted to investigate its effects on protein expressions of key inflammatory targets. For COX-2, iNOS, and mPGES-1 western blots, microglia were left untreated or treated with LPS (100 ng/mL) in the presence or absence of cryptolepine (2.5 and 5 *μ*M) for 24 h. In order to elucidate the effects of cryptolepine on p38 signalling, western blots were also done for phospho-p38 and phospho-MAPKAPK2. In this case, microglia were pretreated for 30 min with cryptolepine (2.5 and 5 *μ*M) and then stimulated for 30 min with LPS (100 ng/mL). At the end of each experiment, cells were washed with phosphate-buffered saline (PBS) and lysed in 1.3 × sodium-dodecyl-sulfate-(SDS-) containing sample buffer without 1, 4-dithio-dL-threitol (DTT) or bromophenol blue containing 100 *μ*M orthovanadate. Protein contents were measured using the bicinchoninic acid method (BCA protein determination kit from Pierce, distributed by KFC Chemikalien, Munich, Germany) according to the manufacturer's instructions. Bovine serum albumin (BSA, Sigma) was used as a standard. Before electrophoresis, bromophenol blue and DTT (final concentration, 10 mM) were added to the samples. For Western blotting, 40 *μ*g of total protein from each sample was subjected to SDS-PAGE (polyacrylamide gel electrophoresis) under reducing conditions. Proteins were then transferred onto polyvinylidene fluoride (PVDF) membranes (Millipore, Bedford, MA, USA) by semi-dry blotting. The membranes were blocked overnight at 4°C using Rotiblock (Roth, Karlsruhe, Germany) and for another hour at room temperature before incubation with the antibodies. Primary antibodies used were goat anti-COX-2 (1 : 500; Santa Cruz), rabbit anti-iNOS (1 : 1000; Cell Signaling) rabbit anti-mPGES-1 (1 : 1000; Agrisera), rabbit anti-phospho-p38 (1 : 1000; Cell Signaling), rabbit anti-phospho-MAPKAPK2 (Assay Biotech, 1 : 1000), and rabbit anti-actin (1 : 5000; Sigma). Primary antibodies were diluted in tris-buffered saline (TBS) containing 0.1% Tween 20 (TBS-T) and 1% BSA. Membranes were incubated with the corresponding primary antibody overnight at 4°C. After extensive washing (three times for 15 min each in TBS-T), proteins were detected with horseradish peroxidase-coupled rabbit anti-goat IgG (Santa Cruz, 1 : 100,000 dilution) or goat anti-rabbit IgG (Amersham, 1 : 25,000 dilution) using chemiluminescence (ECL) reagents (Amersham Pharmacia Biotech, Freiburg, Germany). Equal protein loading and transfer were assessed by subjection of each sample to a Western blot for actin. All Western blot experiments were carried out at least three times.

### 2.5. Quantification of COX-2 and mPGES-1 by Quantitative RT-PCR

In order to further determine effects of cryptolepine on COX-2 and mPGES-1, we evaluated the effects of the compound of their gene expressions. Rat primary microglia were preincubated for 30 minutes with cryptolepine (2.5 and 5 *μ*M); subsequently LPS (100 ng/mL) was added for total 4 h. RNA preparation was done by using RNAspin mini RNA isolation kit (GE Healthcare, Freiburg, Germany) and for cDNA synthesis one microgram of total RNA was reverse transcribed using M-MLV reverse transcriptase and random hexamers (Promega, Mannheim, Germany). The synthesised cDNA was the template for the real-time polymerase chain reaction (PCR) amplification which was carried out by the CFX96 real-time PCR detection system (Bio-Rad Laboratories Inc.) using iQ SYBR Green supermix (Bio-Rad Laboratories GmbH, Munich, Germany). Specific primers were designed by using Primer-Blast (http://www.ncbi.nlm.nih.gov/tools/primer-blast/) program and primers were obtained from Biomers (Ulm, Germany). Reaction conditions were 3 min at 95°C, followed by 40 cycles of 15 s at 95°C, 30 s at 50°C, and 45 sec at 72°C, and every cycle was followed by plate read. After that, reactions conditions were 1 min at 95°C, 1 min at 55°C, followed by melt curve conditions of 65°C–95°C with increment of 0.5°C for 5 sec followed by final plate read. GAPDH served as an internal control for sample normalisation and the comparative cycle threshold Ct method was used for data quantification as described previously [17]. The following primer sequences were used in the present study: COX-2: Fwd 5′-GCCCAGCACTTCACGCATCAGT-3′; Rev 5′-AAGTCCACCCCATGGCCCAGC-3′. mPGES-1: Fwd 5′-TGCAGCACGCTGCTGGTCAT-3′; Reverse 5′-GGCAAAGGCCTTCTTCCGCAG-3′. GAPDH: Fwd 5′-GTCGCCAGCCGAGCCACATC-3′; Reverse: 5′-CCAGGCGCCCAATACGACCA-3′.

### 2.6. Determination of NF-*κ*Bp65 Nuclear Translocation

BV-2 microglia were stimulated with LPS (100 ng/mL) in the presence or absence of cryptolepine for 15 min. After the stimulation period, nuclear extracts were prepared using Cayman nuclear extraction kit (Cayman Chemical Company, Ann Arbor, USA) according to manufacturer's instructions. Briefly, cells were collected by scraping and washed twice with cold PBS. Cells were centrifuged for 5 min at 4°C; the supernatant was discarded and cell pellet was resuspended in 5 mL of ice-cold PBS. The centrifugation procedure was repeated twice, and the cell pellets were placed on ice and were allowed to swell in 500 *μ*L of 1 x Hypotonic Buffer, followed by addition of 10% NP-40 with gentle mixing. The suspension was centrifuged and the supernatants which contained the cytosolic fractions were stored at −80°C for subsequent analysis of cytoplasmic p65 subunit. The pellet was resuspended in 100 *μ*L of ice-cold complete nuclear extraction buffer, vortexed, and rocked gently for 15 min. The samples were then centrifuged and the supernatants (nuclear fractions) were collected. Nuclear fractions were measured for levels of NF-*κ*B using NF-*κ*Bp65 ELISA kit (Invitrogen), according to the manufacturer's instructions.

### 2.7. Determination of I*κ*B Phosphorylation

BV-2 microglia were left untreated or treated with LPS (100 ng/mL) in the presence or absence of cryptolepine (2.5 and 5 *μ*M) for 15 min. At the end of the stimulation period, cells were washed with phosphate-buffered saline (PBS) and lysed with commercially available lysis buffer (New England Biolabs, UK). Cell lysates were subjected to phospho-I*κ*B*α* ELISA according to the manufacturer's instructions (Cell signalling Technology, Inc). Concentrations of phospho-I*κ*B in cell lysates were then measured with a plate reader at 450 nm.

### 2.8. ELISA for MAPK Phosphorylation

BV-2 microglia were left untreated or treated with LPS (100 ng/mL) in the presence or absence of cryptolepine (2.5 and 5 *μ*M) for 30 min. At the end of the stimulation period, cells were washed with phosphate-buffered saline (PBS) and lysed as described for western blot. Cell lysates were subjected to PathScan MAP Kinase Multi-Target Sandwich ELISA for phospho-p38, phospho-42/44, and phospho-JNK according to the manufacturer's instructions (Cell Signalling Technologies). Absorbance values were measured with a plate reader at 450 nm.

### 2.9. MTT Assay for Cell Viability

The viability of BV-2 cells after treatment with cryptolepine was determined by the colorimetric 3-(4, 5-dimethylthiazol-2-yl)-2,5-diphenyl tetrazolium bromide (MTT) assay. The yellow compound MTT is reduced by mitochondrial dehydrogenases to the water-insoluble blue compound formazan, depending on the viability of cells. BV-2 cells (2 × 10^5^/200 *μ*L/well) were cultured for 2 days and then incubated with or without LPS (100 ng/mL) in the absence or presence of cryptolepine (2.5 and 5 *μ*M) for 24 h. Twenty microlitres MTT solution (Sigma) (5 mg/mL) were added to each well. The plate was incubated for 4 h at 37°C in a CO_2_ incubator. One hundred and eighty microlitres of medium was removed from every well without disturbing the cell clusters. A 180 *μ*L methanol/DMSO solution (50 : 50) was added to each well, and the preparations were mixed thoroughly on a plate shaker with the cell containing formazan crystals. After all of the crystals were dissolved, the absorbance was read at 540 nm with a microplate reader.

### 2.10. Statistical Analysis

Data from at least three independent experiments were used for analysis. Mean ± SEM was calculated. Values were compared using one-way ANOVA with post hoc Student Newman–Keuls test (multiple comparisons). The level of significance was set at *P* < 0.05.

## 3. Results

### 3.1. Cryptolepine Reduced the Production of TNF*α*, IL-1*β*, and IL-6 from LPS-Activated Microglia

**Figure 2:**
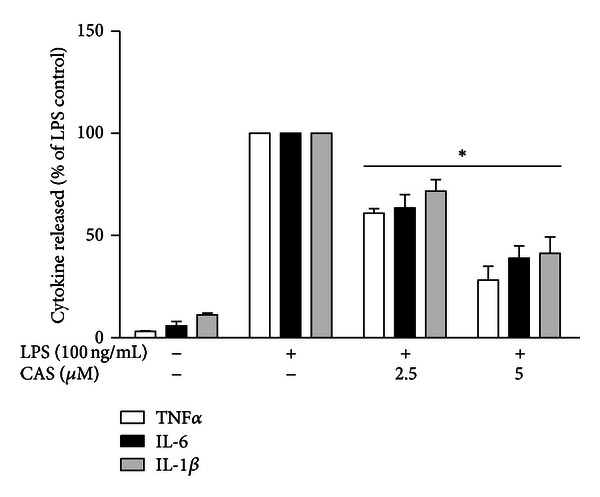
Cryptolepine (CAS) reduced TNF*α*, IL-6, and IL-1*β* production in LPS-activated microglia. Cells were stimulated with LPS (100 ng/mL) in the presence or absence of CAS (2.5 and 5 *μ*M) for 24 h. At the end of the incubation period, supernatants were collected for ELISA measurements. All values are expressed as mean ± SEM for 3 independent experiments. Data were analysed using one-way ANOVA for multiple comparison with post hoc Student Newman-Keuls test. *P < 0.05 in comparison with LPS control.

To determine the effects of cryptolepine on the production of mediators of neuroinflammation, we investigated its effects on LPS-activated primary microglia. Cells were preincubated with 2.5 and 5 *μ*M of cryptolepine for 30 min and then stimulated with LPS (100 ng/mL) for 24 h; cytokine levels were then determined using ELISA. As shown in Figure 2, levels of TNF*α*, IL-6, and IL-1*β* were barely undetectable in the control cells. However, LPS produced marked production of all cytokines, while pretreatment with 2.5 and 5 *μ*M resulted in significant (*P* < 0.05) reduction in the production of TNF*α*, IL-6, and IL-1*β*.

### 3.2. Cryptolepine Suppressed PGE_2_ Production by Inhibiting COX-2 and mPGES-1 Protein and Gene Expressions in LPS-Activated Microglia

**Figure 3:**
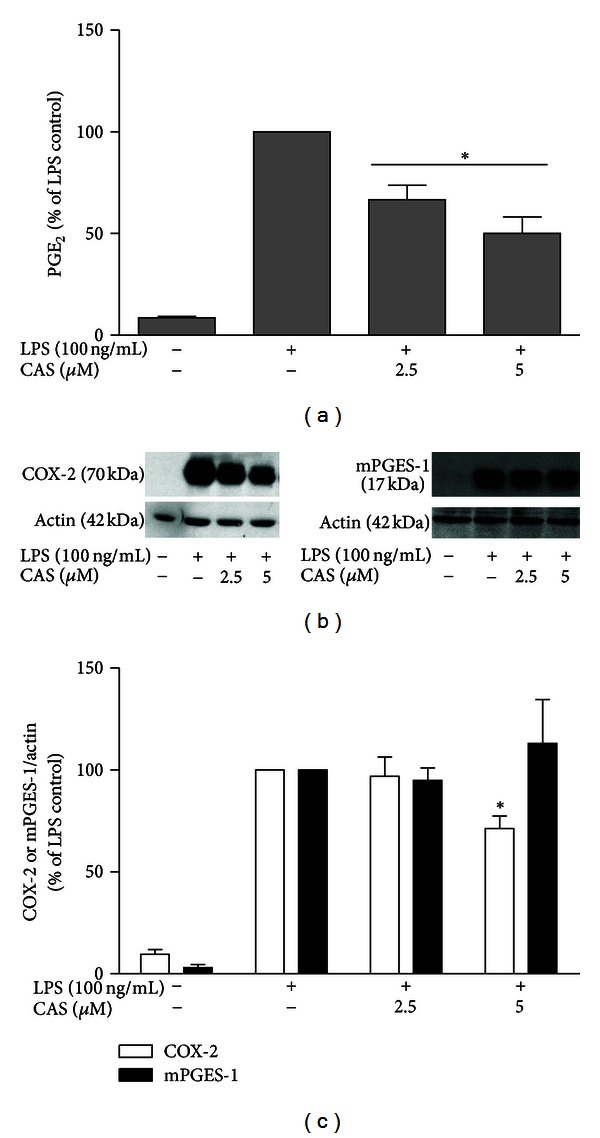
Cryptolepine (CAS) inhibited PGE_2_ release and COX-2 protein expressions in LPS-activated microglia. Microglia were incubated in a medium containing 2.5 and 5 *μ*M cryptolepine for 30 min and then activated by 100 ng/mL LPS for 24 h. (a) Cryptolepine diminished PGE_2_ release in microglia. ((b), (c)) Cryptolepine inhibited COX-2 but not mPGES1 protein expression in LPS-activated microglia. Protein expression was determined using western blot with specific anti-COX-2 and anti-mPGES-1 antibodies. All values are expressed as mean ± SEM for 3 independent experiments. Data were analysed using one-way ANOVA for multiple comparisons with post-hoc Student Newman-Keuls test. *P < 0.05 in comparison with LPS control.

The ability of cryptolepine to reduce the production of PGE_2_ was investigated using primary microglial cells stimulated with LPS. Activation of microglial cells produced marked increase in the production of PGE_2_ after 24 h of incubation with LPS (Figure 3(a)). Pretreatment with cryptolepine resulted in a significant reduction in the formation of PGE_2_ in the cell supernatants.

Following our findings that cryptolepine significantly attenuated LPS-induced PGE_2_ production, we further investigated the effects of the compound on COX-2 protein and mRNA expressions in rat microglia.

**Figure 4:**
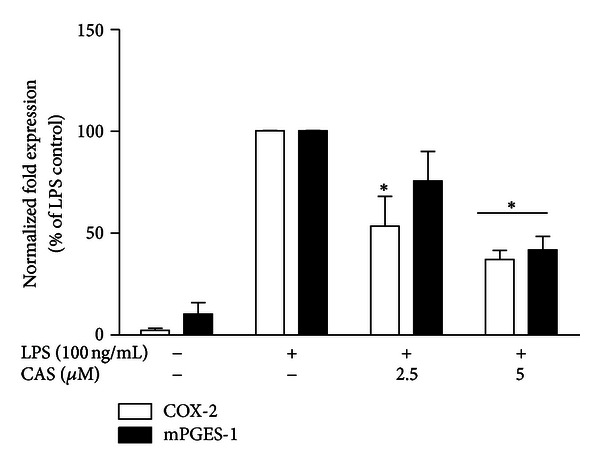
Cryptolepine (CAS) inhibited the increase in COX-2 and mPGES-1 mRNA in LPS-stimulated microglia. Cells were stimulated with LPS (100 ng/mL) in the presence or absence of CAS (2.5 and 5 *μ*M) for 4 h. At the end of incubation periodtotal RNA was extracted and subjected to RT-PCR. All values are expressed as mean ± SEM for 3 independent experiments. Data were analysed using one-way ANOVA for multiple comparisons with post hoc Student Newman-Keuls test. *P < 0.05 in comparison with LPS control.

Figures 3(b) and 3(c) show that stimulation of microglia with LPS produced marked expression of COX-2 protein. At 2.5 *μ*M, cryptolepine did not produce a significant effect on LPS-induced COX-2 protein expression. However, pretreatment with 5 *μ*M of the compound resulted in significant reduction in COX-2 protein expression following LPS activation. Interestingly, both concentrations of cryptolepine significantly suppressed COX-2 gene expression in LPS-activated microglia (Figure 4).

The microsomal prostaglandin E_2_ synthase (mPGES-1) is the terminal enzyme in the production of PGE_2_; it is induced by proinflammatory stimuli and functionally coupled with COX-2 in marked preference to COX-1. Consequently, we decided to determine if mPGES-1 contributed to the effects of cryptolepine on PGE_2_ production in LPS-activated microglia. Results show that cryptolepine (2.5 and 5 *μ*M) did not reduce mPGES-1 protein expression (Figures 3(b) and 3(c)). At 2.5 *μ*M, the effect of cryptolepine on mPGES-1 gene expression was not significant. However, pretreatment with 5 *μ*M produced significant downregulation of mPGES-1 gene in LPS-activated microglia (Figure 4).

### 3.3. Inhibition of NO/iNOS Contributes to the Anti-Inflammatory Action of Cryptolepine in LPS-Activated Microglia

**Figure 5:**
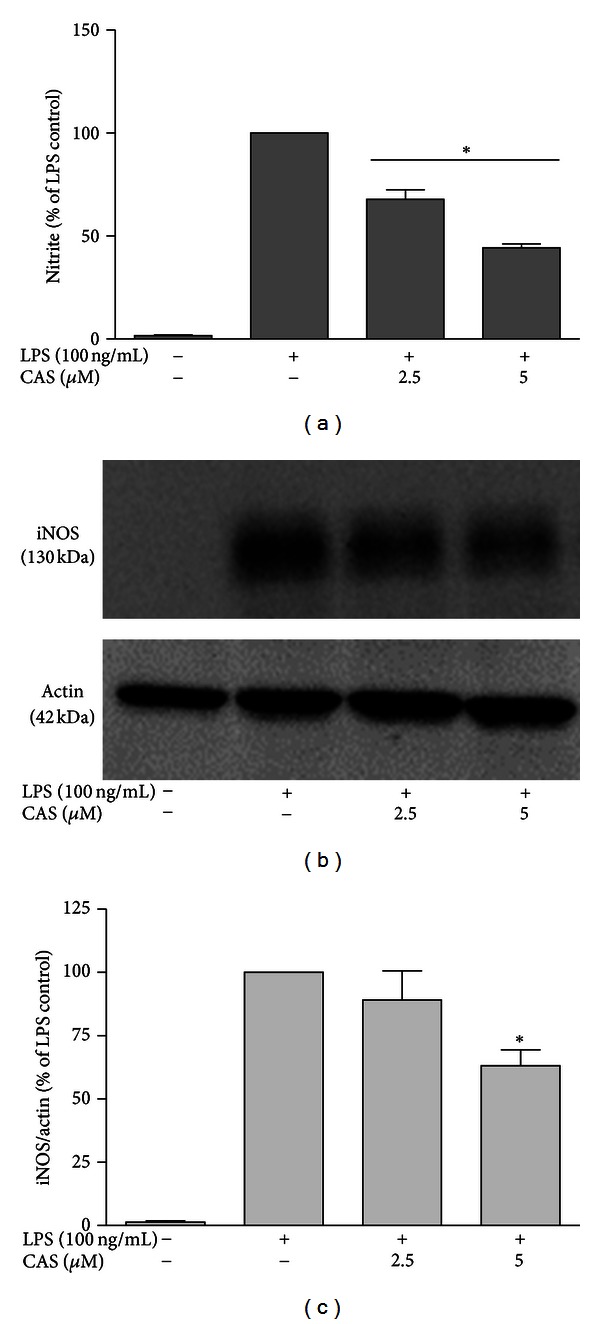
Cryptolepine (CAS) inhibited nitrite release and iNOS protein expression in LPS-activated microglia. Microglia were incubated in a medium containing 2.5 and 5 *μ*M cryptolepine for 30 min and then activated by 100 ng/mL LPS for 24 h. (a) Cryptolepine diminished nitrite release in microglia. ((b), (c)) Cryptolepine inhibited iNOS protein expression in LPS-activated microglia. Protein expression was determined using western blot with specific anti-iNOS antibody. All values are expressed as mean ± SEM for 3 independent experiments. Data were analysed using one-way ANOVA for multiple comparisons with post hoc Student Newman-Keuls test. *P < 0.05, in comparison with LPS control.

To investigate the effects of cryptolepine on NO production in LPS-stimulated microglial cells, cells were treated with LPS alone or with 2.5 and 5 *μ*M of cryptolepine for 24 h. The levels of NO in the culture media were determined with the Griess assay. Cryptolepine (2.5 and 5 *μ*M) significantly (*P* < 0.05) decreased the LPS-induced production of NO in microglial cells (Figure 5(a)). Next, to elucidate the mechanism responsible for the inhibitory effect of cryptolepine on NO production, we determined the iNOS protein levels with immunoblotting analysis. Cryptolepine (5 *μ*M) significantly (*P* < 0.05) inhibited iNOS protein expression in the rat microglial cells (Figures 5(b) and 5(c)).

### 3.4. Anti-Inflammatory Action of Cryptolepine is Mediated by Inhibition of Phosphorylation of p38 and Its Downstream Kinase MAPKAPK2 in the Microglia

**Figure 6:**
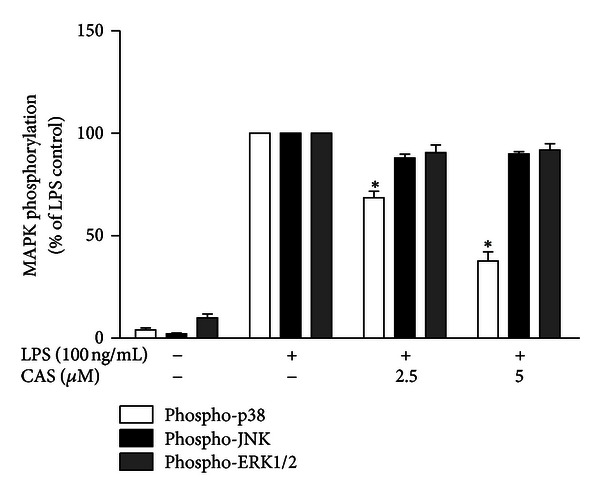
Cryptolepine (CAS) inhibited p38 but not p42/44 or JNK MAPK in LPS-activated BV-2 microglia. Cells were stimulated with LPS (100 ng/mL) in the presence or absence of CAS (2.5 and 5 *μ*M) for 30 min. Lysates from activated microglia were then analysed using sandwich ELISA for phospho-p38, phospho-42/44 and phospho-JNK. Data were analysed using one-way ANOVA for multiple comparisons with post hoc Student Newman-Keuls test. *P < 0.05 in comparison with LPS control.

We next determined whether the suppressive effect of cryptolepine on synthesis and release of proinflammatory mediators occurred via MAPK signalling pathway. BV2 cells were treated with 2.5 and 5 *μ*M cryptolepine in the presence or absence of LPS for 30 min, and levels of MAPK were determined by ELISA. As shown in Figure 6, cryptolepine did not inhibit LPS-induced phosphorylation levels of ERK1/2 and JNK MAPKs, while LPS-induced phosphorylation of p38 MAPK was significantly inhibited by cryptolepine in BV2 microglia.

**Figure 7:**
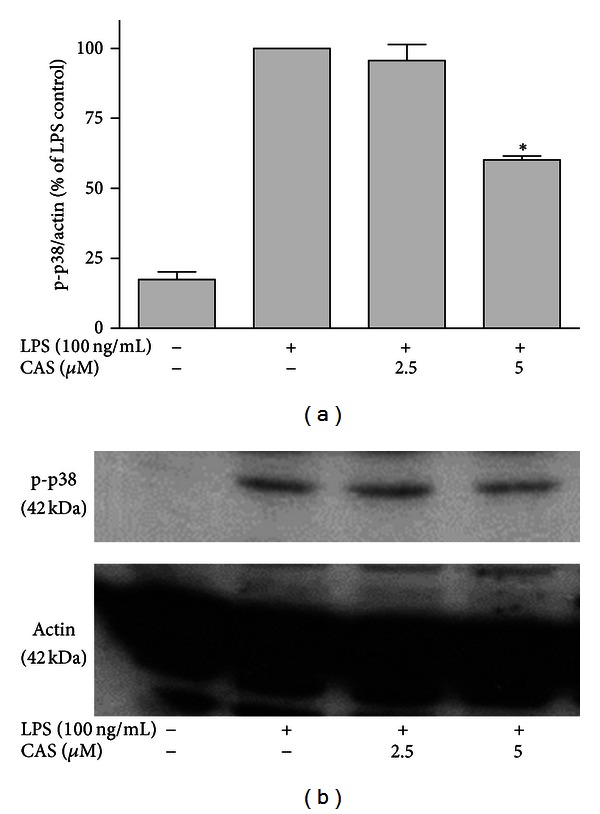
Cryptolepine (CAS) inhibited p38 phosphorylation in LPS-activated microglia. Cells were stimulated with LPS (100 ng/mL) in the presence or absence of CAS (2.5 and 5 *μ*M) for 30 min. At the end of incubation period, phospho-p38 protein expression was determined using western blot with specific anti-phospho-p38 antibody. All values are expressed as mean ± SEM for 3 independent experiments. Data were analysed using one-way ANOVA for multiple comparison with post hoc Student Newman-Keuls test. *P < 0.05 in comparison with LPS control.

To confirm the previous result, immunoblotting for phosphorylation of p38 MAPK was also carried out in primary microglial cells. The results showed that on treatment with LPS, there was an increased expression of phospho-p38 MAPK protein, which was significantly (*P* < 0.05) reduced by 5 *μ*M of cryptolepine (Figures 7(a) and 7(b)). These findings indicate that cryptolepine is effective in the inhibition of p38 MAPK phosphorylation in LPS-stimulated microglial cells.

**Figure 8:**
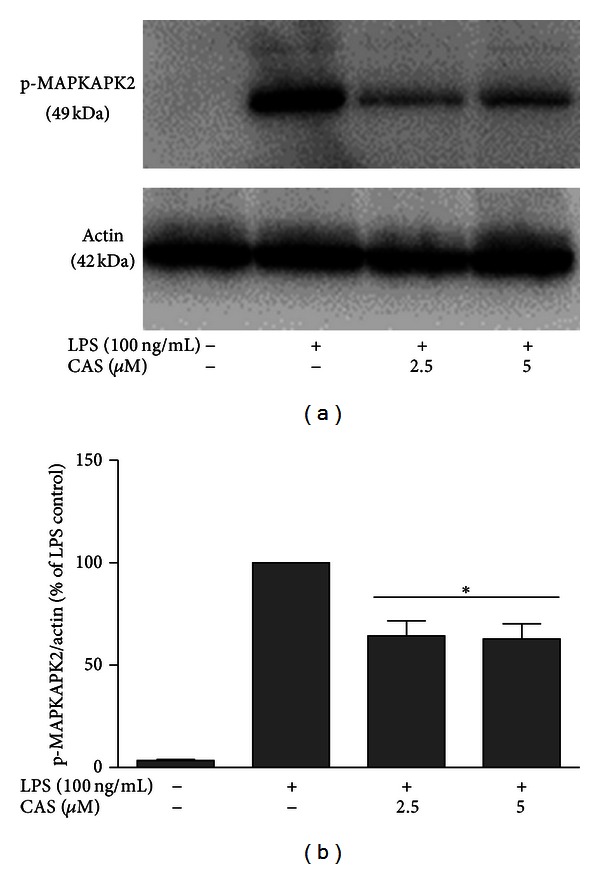
Cryptolepine (CAS) inhibited MAPKAPK2 phosphorylation in LPS-activated microglia. Cells were stimulated with LPS (100 ng/mL) in the presence or absence of CAS (2.5 and 5 *μ*M) for 30 min. At the end of incubation period, phospho-MAPKAPK2 protein expression was determined using western blot with specific anti-phospho-MAPKAPK2 antibody. All values are expressed as mean ± SEM for 3 independent experiments. Data were analysed using one-way ANOVA for multiple comparison with post hoc Student Newman-Keuls test. *P < 0.05, in comparison with LPS control.

As MAPKAPK2 is an immediate downstream substrate of p38 MAPK, we also investigated whether this kinase is also targeted by cryptolepine.  Figure 8 shows that LPS stimulation produced MAPKAPK2 phosphorylation, which was significantly inhibited with cryptolepine (2.5 and 5 *μ*M) pretreatment. This indicates that cryptolepine blocks direct phosphorylation of MAPKAPK2 by p38 MAPK to produce anti-inflammatory action in LPS-activated microglia.

### 3.5. Cryptolepine Inhibits Nuclear Translocation of NF-*κ*B but Not I*κ*B Phosphorylation in Microglia

**Figure 9:**
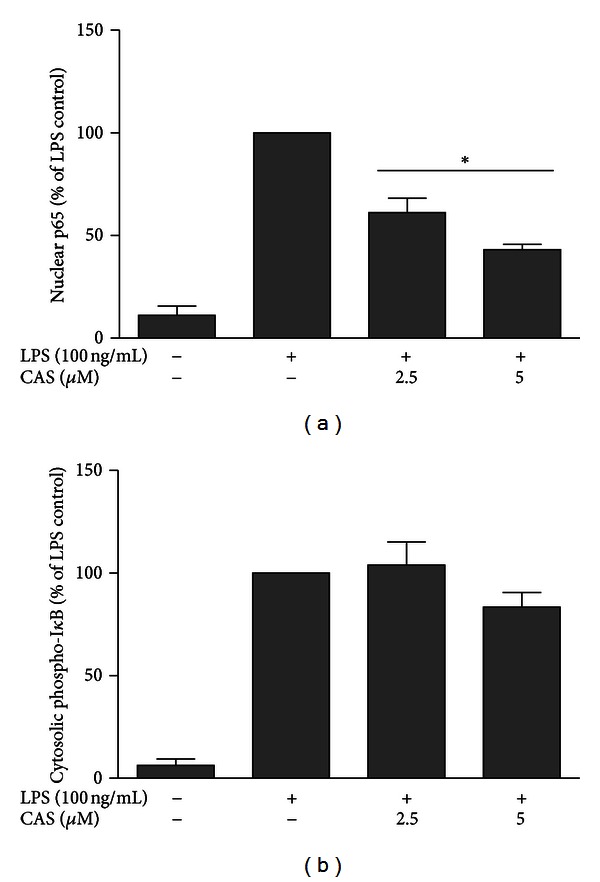
Cryptolepine (CAS) inhibited LPS-induced nuclear translocation of NF-*κ*B (a), but not I*κ*B phosphorylation (b) in LPS-activated microglia. Cells were stimulated with LPS (100 ng/mL) in the presence or absence of CAS (2.5 and 5 *μ*M) for 15 min. Nuclear extracts were analysed for levels of NF-*κ*Bp60, while lysates were analysed for phospho-I*κ*B using an ELISA kit. All values are expressed as mean ± SEM for 3 independent experiments. Data were analysed using one-way ANOVA for multiple comparison with post hoc Student Newman-Keuls test. *P < 0.05 in comparison with LPS control.

LPS is known to produce neuroinflammatory-associated gene expression through the activation of NF-*κ*B. Based on the inhibition of neuroinflammatory molecules in microglia by cryptolepine, we used ELISA to test the effects of the compound on LPS-induced translocation of the NF-*κ*B p65 subunit in the microglia. Our experiments showed that treatment with LPS induced translocation of the p65 subunit from the cytosol to the nucleus. This process was significantly inhibited by both concentrations of cryptolepine tested (Figure 9).

**Figure 10:**
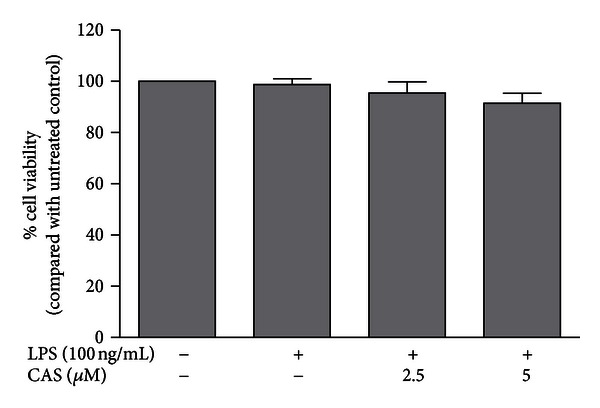
Pretreatment with 2.5 and 5 *μ*M of cryptolepine (CAS) did not affect the viability of BV-2 microglia stimulated with 100 ng/mL LPS.

Based on the observation that cryptolepine inhibited nuclear translocation of NF-*κ*B, we next assessed if this effect was related to phosphorylation of I*κ*B. Surprisingly, we found that cryptolepine did not produce significant inhibition of I*κ*B phosphorylation in LPS-stimulated microglia (Figure 10), suggesting that the compound might be acting on NF-*κ*B through I*κ*B-independent mechanism(s).

### 3.6. Anti-Inflammatory Concentrations of Cryptolepine Did Not Affect Viability of Microglia

In order to determine the effects of cryptolepine on cell viability, an MTT assay was done on BV-2 microglia. Figure 10 shows that cryptolepine (2.5 and 5 *μ*M) treatment did not produce significant effect on cell viability.

## 4. Discussion

It is increasingly evident that neuroinflammatory mechanisms are implicated in the pathogenesis of neurodegenerative disorders like AD. Furthermore, evidence has demonstrated sustained inflammatory responses involving microglia and astrocytes in animal models of neurodegeneration [18].

Cryptolepine has been widely reported to exhibit anti-inflammatory activity. A study by Bamgbose and Noamesi [19] reports that cryptolepine (1, 5, 10, and 20 mg/kg) inhibited carrageenan-induced rat paw oedema. This observation was subsequently confirmed by a study which also describes the effect of cryptolepine on carrageenan-induced pleurisy and lipopolysaccharide-induced microvascular permeability in mice [13]. Earlier, we showed that cryptolepine inhibited NO production and DNA binding of NF*κ*B in LPS-stimulated RAW 264.7 macrophages [12]. In the present study, we investigated the effects of cryptolepine on neuroinflammation in the microglia following activation by LPS.

Microglial cells are known to release proinflammatory cytokines such as IL-1, IFN*γ*, IL-6, and TNF*α*, when activated [20]. Activated microglia have also been reported to produce potentially neurotoxic substances like nitric oxide, oxygen radicals, and proteolytic enzymes, as well as pro-inflammatory cytokines [21]. Consequently, we investigated whether cryptolepine had an effect on the production of TNF*α*, IL6, and IL1*β* in LPS stimulated microglial cells. Our results indicate that micromolar concentrations of cryptolepine significantly suppress the production of these cytokines in the activated microglia.

Our data show that cryptolepine inhibits PGE_2_ production as well as COX-2 protein and gene expressions in LPS-treated microglia cells. PGE_2_ is an arachidonic acid derived proinflammatory mediator released by microglia [22]. 

Evidence indicates that mPGES-1 is inducible in various models of pain and inflammation, where it is the predominant synthase involved in COX-2-mediated PGE_2_ production [23]. In the biosynthetic pathway resulting in PGE_2_ production, arachidonic acid is converted to PGH_2_ by COX-1 or COX-2 and is then converted to PGE_2_ by prostaglandin E synthases. Interestingly, our data show that cryptolepine suppressed gene, but not protein expression of mPGES-1. A study has suggested that the regulation of mPGES-1 and COX-2 is not strictly coupled to each other in the microglia [24]. Also, PGE_2_ production has been shown to be unaffected by silencing mPGES-1 in IL-1*β* or TNF-*α*-stimulated gingival fibroblasts [25]. Therefore, we hypothesise that the effect of cryptolepine on COX-2 expression in LPS-activated microglia is probably independent of mPGES-1 and that there might be other mechanisms contributing to the effects of cryptolepine on COX-2.

Mouse and rat microglia have been shown as potent producers of NO upon activation with LPS and/or pro-inflammatory cytokines [26]. In this study, we demonstrated that LPS induces an increase in iNOS immunoreactivity and NO release in microglial cells, which were inhibited by cryptolepine.

LPS and other inflammatory stimuli have been reported to activate the MAPK and NF-*κ*B signalling pathways in microglia [27]. In particular, NF-*κ*B is an important upstream regulator of cytokine, COX-2, and iNOS expressions [28]. Studies have also shown that blockade of NF-*κ*B transcriptional activity in the CNS can suppress expression of iNOS, COX-2, and the proinflammatory cytokines, such as IL-1*β*, IL-6, and TNF*α* [29]. It is also widely known that LPS stimulation increases NF-*κ*B activation through I*κ*B*α* phosphorylation and degradation, leading to nuclear translocation of the p65 subunit. Consequently, we carried out ELISAs to determine whether pretreatment with cryptolepine inhibited LPS-induced NF-*κ*B activation and I*κ*B*α* phosphorylation in the microglia. Our data clearly demonstrate that LPS treatment caused activation and nuclear translocation of p65 subunit in the microglia; this phenomenon was statistically inhibited by cryptolepine. Interestingly, inhibition of LPS-stimulated I*κ*B phosphorylation was not achieved with cryptolepine pretreatment. Earlier, we showed that cryptolepine inhibited activation and DNA binding of NF-*κ*B without affecting I*κ*B phosphorylation in RAW 264.7 macrophages [12]. This study seems to confirm that cryptolepine might be acting on NF-*κ*B translocation and/or nuclear binding by unknown mechanism(s) independent of I*κ*B phosphorylation and subsequent degradation.

Studies have demonstrated that the MAPKs (p38, JNK, ERK1/2) were activated in both glia and neurons following LPS treatment, which suggested their involvement in both glial activation and neuronal response to glia-derived neurotoxic molecules. Pharmacological inhibition studies further demonstrated that p38 and JNK MAPKs, but not ERK1/2 MAPK, are important signal transduction pathways contributing to glia-induced neuron death [30, 31]. The results of our studies show that cryptolepine inhibited LPS-induced p38, but not JNK or ERK1/2 phosphorylation in the microglia. This clearly shows that suppression of p38 phosphorylation is critical to the anti-inflammatory action of cryptolepine in LPS-stimulated microglia. This prompted us to examine the effect of the compound on the downstream substrate of p38, MAPKAPK2 (MK2). MK2 activation and expression have been shown to be increased in microglial cells stimulated with LPS and gamma interferon [11]. Cryptolepine significantly inhibited phosphorylation of MK2 which resulted from activation of microglia by LPS. This suggests that anti-inflammatory action of cryptolepine in activated microglia is strongly linked to its influence on p38 MAPK signalling. 

## 5. Conclusions

In this study, we have provided evidence to show that cryptolepine produces anti-inflammatory effects in LPS-activated microglia by targeting PGE_2_/COX-2 production. We also demonstrated that this compound might owe its anti-inflammatory activity to I*κ*B-independent events in NF-*κ*B signalling which are not fully understood. However, it was demonstrated that cryptolepine strongly inhibits p38/MK2 signalling, which might be critical in further studies on the anti-neuroinflammatory property of the compound.
